# Barriers and facilitators to clinical implementation of radiotherapy treatment planning automation: A survey study of medical dosimetrists

**DOI:** 10.1002/acm2.13568

**Published:** 2022-03-03

**Authors:** Rachel Petragallo, Naomi Bardach, Ezequiel Ramirez, James M. Lamb

**Affiliations:** ^1^ Department of Radiation Oncology University of California Los Angeles California USA; ^2^ Department of Pediatrics University of California San Francisco California USA; ^3^ Department of Radiation Oncology University of California San Francisco California USA

**Keywords:** auto‐contouring, automated treatment planning, implementation science, medical dosimetrists, survey study

## Abstract

**Purpose:**

Little is known about the scale of clinical implementation of automated treatment planning techniques in the United States. In this work, we examine the barriers and facilitators to adoption of commercially available automated planning tools into the clinical workflow using a survey of medical dosimetrists.

**Methods/materials:**

Survey questions were developed based on a literature review of automation research and cognitive interviews of medical dosimetrists at our institution. Treatment planning automation was defined to include auto‐contouring and automated treatment planning. Survey questions probed frequency of use, positive and negative perceptions, potential implementation changes, and demographic and institutional descriptive statistics. The survey sample was identified using both a LinkedIn search and referral requests sent to physics directors and senior physicists at 34 radiotherapy clinics in our state. The survey was active from August 2020 to April 2021.

**Results:**

Thirty‐four responses were collected out of 59 surveys sent. Three categories of barriers to use of automation were identified. The first related to perceptions of limited accuracy and usability of the algorithms. Eighty‐eight percent of respondents reported that auto‐contouring inaccuracy limited its use, and 62% thought it was difficult to modify an automated plan, thus limiting its usefulness. The second barrier relates to the perception that automation increases the probability of an error reaching the patient. Third, respondents were concerned that automation will make their jobs less satisfying and less secure. Large majorities reported that they enjoyed plan optimization, would not want to lose that part of their job, and expressed explicit job security fears.

**Conclusion:**

To our knowledge this is the first systematic investigation into the views of automation by medical dosimetrists. Potential barriers and facilitators to use were explicitly identified. This investigation highlights several concrete approaches that could potentially increase the translation of automation into the clinic, along with areas of needed research.

## 1 INTRODUCTION

In recent years there has been a substantial increase in research and development involving automation of the radiation therapy treatment planning workflow. Treatment planning automation can be used to reduce the occurrence of suboptimal treatment plan quality,^1^ facilitate adaptive radiotherapy,^2^ reduce treatment latency, and allow human cognitive resources to be directed to their most high value uses. Almost every step of the radiation treatment workflow, from normal tissue contouring,^3^, ^4^ to intensity‐modulated radiotherapy treatment planning,^5^, ^6^ to online adaptive replanning,^7^ to the physics plan review process,^8^, ^9^, ^10^ has been subject to automation research. Automated normal tissue contouring tools have demonstrated accuracy comparable to manual contours for many target organs,^11^ and demonstrated significant time savings in clinical workflow studies.^12^, ^13^ A wealth of research has demonstrated that automated radiotherapy planning techniques are capable of producing clinical‐quality plans.^14^, ^15^ Prospective studies have shown that in carefully controlled situations, automated treatment planning (ATP) generates plans of comparable or better clinical quality, at significant time savings.^16^, ^17^, ^18^ A variety of commercially available automation tools exist, including but not limited to atlas‐based^19^ and deep‐learning‐based^13^ auto‐contouring (AC), knowledge‐based planning (KBP),^20^ rule‐based automated planning,^21^ and automated field‐in‐field planning.^22^

However, little is known about the scale of clinical implementation of ATP techniques in the United States. We hypothesized that clinical adoption of treatment planning automation may be less than fully realized and furthermore that barriers to the implementation may exist. These hypotheses were based on the reported observation of barriers to automation in similar areas of healthcare such as diagnostic radiology^23^, ^24^ and pharmacy.^25^ Additionally, the authors have previously collected anecdotal data that medical dosimetrists, who in many clinics perform the majority of treatment planning, often express hesitation at using treatment planning automation. In order to ensure that advances in research translate into advances in clinical care, a focused effort is required in order to understand the barriers to implementation.^26^ While an individual clinic may be committed to the adoption of evidence‐based best practices in principle, the actual implementation of these practices requires a thorough understanding of all the breakpoints where such implementation can fail.^27^ The diversity of health care settings in the United States represents a major challenge to the widespread dissemination of evidence‐based best practices.^28^

In this work, we examine the barriers and facilitators to adoption of commercially available automatic treatment planning tools into the clinical workflow using a survey of medical dosimetrists. We focus on how implementation of treatment planning automation is viewed by medical dosimetrists within the radiation oncology clinic. Here we define treatment planning automation as the automation of parts of the treatment planning workflow, such as AC and automated dose optimization. To our knowledge, complete end‐to‐end automation of the treatment planning workflow has very limited if any clinical implementation, but our survey left open the possibility for respondents to address complete automation as well. To date, no published research has examined whether or why medical dosimetrists may view these tools favorably or unfavorably. Our primary goal is to identify the barriers to implementation from the perspective of the medical dosimetrist. Our secondary goal is to offer potential facilitators to increase the adoption of evidence‐based best practices with respect to ATP in the context of the radiation oncology clinic.

## 2 METHODS AND MATERIALS

### 2.1 Survey design

Survey questions broadly probed the following areas: frequency of use of treatment planning automation (AC and automated dose optimization), positive and negative perceptions about automation performance, potential implementation changes that would affect accessibility and usability, and demographics and institutional descriptive statistics. Positive and negative questions were balanced to reduce bias.^29^ Level of agreement questions were heavily utilized because they facilitate balanced positive and negative statements.

Several of the best practices compiled by Krosnick^30^ in his 1999 review paper are worth briefly mentioning as they are relevant to our own survey methodology. First, in order to draw general conclusions about a population based on survey responses from a sample, it is imperative to ensure a representative sample has been obtained. Our survey measures familiarity with and attitudes toward automation, but we were careful not to restrict potential respondents based on their prior use of these tools. Instead, the only criteria we imposed was a sampling of dosimetrists currently employed in our state, regardless of their prior experience with automation in their workplace. Second, we employed close‐ended questions throughout our survey. Close‐ended questions can be used effectively when the choices given constitute a comprehensive list of all possible options.^30^ In all of our survey questions, great care was taken to ensure that respondents were presented with a comprehensive list of all possible options. Third, all points on rating scale questions were fully labeled and intended to divide the response continuum into approximately equal intervals in order to maximize validity.^31^ It is well‐documented that respondents have a tendency to place themselves toward the middle when answering rating scale questions^30^; however this phenomenon was not observed at large in our data. Finally, research has shown a tendency for respondents to agree with statements more frequently than they disagree.^30^ While we did employ the frequent use of “agree/disagree” question formats, care was taken to ensure that we maintained a balance between positive and negative descriptions of automation.

Cognitive interviews were used to refine an initial survey draft. Cognitive interviewing is a well‐established technique for improving survey quality^32^, ^33^ and is essential for ensuring that survey questions are interpreted correctly and consistently by respondents. Four medical dosimetrists from our department completed the survey on their own time and then were interviewed in detail about their responses. Feedback from these interviews was compiled and incorporated into the final survey. Several questions were omitted from the final survey due to being viewed as too general or not relevant. The wording was modified on several additional questions in order to clarify the meaning and ensure that respondents were consistent in their interpretation. Finally, the choice to incorporate branching logic into the survey design was made following the cognitive pretesting process. The initial survey included questions to gauge favorable or unfavorable views of ATP in general, but it became apparent following the cognitive interviews that this was too broad. Volunteers viewed some automated tools favorably and others unfavorably, with the net result being that they selected a neutral response. Therefore, the choice was made to incorporate branching logic and ask respondents to answer questions regarding each specific automated planning tool they had used.

The final survey questions can be broken down into five general subsections: prior use, AC, ATP, general level of agreement, and demographics. The prior use section consisted of a single question that asked respondents to mark any specific automation tool they had used at any point during their career. Table 1 lists the categories of treatment planning automation delineated in this question, along with the commercial product names used as examples of each category. Responses to this question determined the branching logic that would follow for the remainder of the survey. In the AC section, respondents were asked questions to gauge their level of experience with AC tools, what types of commercially available tools they have used, what anatomic sites they have used AC for, and reasons why they view the tools they have used favorably or unfavorably. The ATP section asked respondents to answer questions regarding their level of experience with ATP and how often they use it, what types of ATP algorithms they have used, what anatomic sites they have used ATP for, and reasons why they like or dislike the ATP tools that they have experience using. Branching logic was used to only show survey questions relevant to the specific AC and/or ATP tools that the participant had prior experience using. The general level of agreement section consisted of a list of statements designed to elicit responses on how participants view automation in ways that may not be specific to individual automation tools. The demographics section consisted of questions about the participant's age, gender, length of time employed in the field, education and relevant certifications, and current clinical environment. The survey was deployed using the secure survey platform Qualtrics. The complete list of survey questions can be found in (Supporting Information).

**TABLE 1:** Example products for each category of auto‐contouring (AC) and automated treatment planning (ATP) surveyed

AC/ATP category	Example products
Deep learning‐based AC	Mirada DLCExpert, MIM ContourProtege AI
Atlas‐based and/or model‐based AC	MIM Atlas Segment, Varian Velocity, RayStation MABS/MBS, Pinnacle SPICE, Elekta ABAS
Knowledge‐based plan quality assessment	Sun Nuclear PlanIQ
Automated planning using knowledge‐based planning algorithms	Varian RapidPlan
Automated field‐in‐field planning	Radformation EZFluence
Automated planning using rule‐based or template‐based algorithms	Philips Pinnacle Auto‐Planning, Raysearch Raystation Auto‐Planning

### 2.2 Recruitments of subjects

Subjects were recruited through two different channels in accordance with the approved Institutional Review Board protocol. The authors used LinkedIn to solicit responses from medical dosimetrists currently employed in a clinical capacity within the state of California. All medical dosimetrists with LinkedIn accounts showing employment as clinical dosimetrists in California were contacted directly on LinkedIn. Additionally, professional contacts of the investigators employed outside of their department were recruited. Finally, all chief physicists of non‐academic medical centers (AMCs) in California listed in the American Association of Physicists in Medicine (AAPM) member directory were contacted with requests for references to medical dosimetrists. This was done in an attempt to balance the responses in a way that more accurately reflected the distribution of academically and non‐academically employed dosimetrists in California. No participant was recruited with whom there was a supervisorial relationship with any of the authors. During the recruitment process, we did not require familiarity with or regular use of ATP as a prerequisite for survey participation. In the event of a non‐response from a potential participant, the authors reached out a second time but did not aggressively pursue a higher response rate beyond this second contact. It has been shown that a low response rate does not inherently indicate the presence of non‐response error in the final data.^34^, ^35^

### 2.3 Statistical analysis

Fisher's exact test was used to test for statistical significance of correlations between measures of use of automation with demographic variables. Clustering analysis was performed in the R programming environment^36^ in order to identify latent groups in the responses. All figures were produced using Python's data visualization package matplotlib.^37^

## 3 RESULTS

**FIGURE 1:**
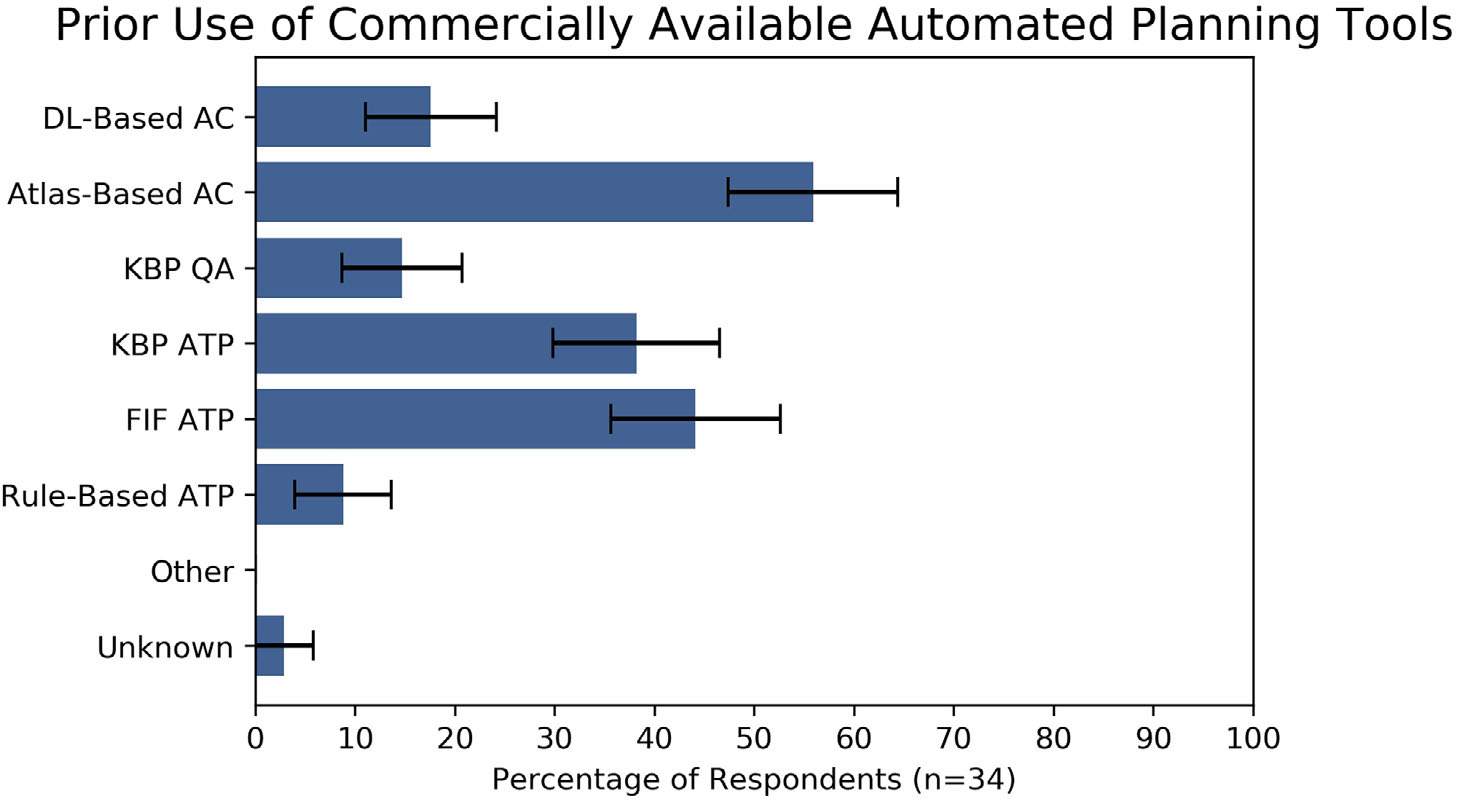
Reported frequency of use of commercially available automated tools. Error bars represent one standard error Abbreviations: AC, auto‐contouring; ATP, automated treatment planning; DL, deep learning; FIF, field‐in‐field; KBP, knowledge‐based planning.

In total 171 medical dosimetrists were contacted either on LinkedIn or by email, of which 57 responded and were sent a survey link. Of the dosimetrists who were sent a survey link, 34 completed the survey. Clinical use of AC remains limited, with 70.6% of respondents (24/34) reporting that they used AC less than weekly. Use of ATP was more frequent, with 41.2% reporting that they used it at least weekly. Respondents reported approximately equal familiarity with AC and ATP, with average familiarity scores of 2.82 and 2.59 out of 5 for AC and ATP, respectively. Despite recent research demonstrating that deep‐learning‐based AC is more accurate than atlas‐based AC, most respondents reported that they used atlas‐based AC. Use of ATP algorithms was more heterogeneous, and the most commonly used algorithm was automated field‐in‐field planning (see Figure 1).

Respondents were more likely (18/34) to have heard the most about AC from scientific talks and vendor booths at professional meetings, and more likely (22/34) to have heard about it least frequently from peers at their own clinic or elsewhere. Survey results broadly sampled level of education, gender, place of employment (academic vs. non‐academic hospital vs. community clinic), but appeared to be weighted toward relatively young dosimetrists, with 61.8% of respondents reporting ages less than 39 years. Complete demographics are contained in Table 2.

**TABLE 2:** Survey respondent demographics

Survey respondent demographics (*n* = 34)	Responses
1. Age (years)	
20–29	4
30–39	17
40–49	5
50–59	7
60+	1
2. Years of experience	
<5	11
5–9	11
10–19	9
20+	3
3. Gender	
Male	18
Female	16
4. Level of education	
Associate's degree	4
Bachelor's degree	16
Master's degree	13
Doctorate	1
5. Place of employment	
Academic medical center	16
Non‐academic hospital	12
Community practice	6
6. Number of radiotherapy machines	
1	3
2–4	14
5–8	10
9+	7

**FIGURE 2:**
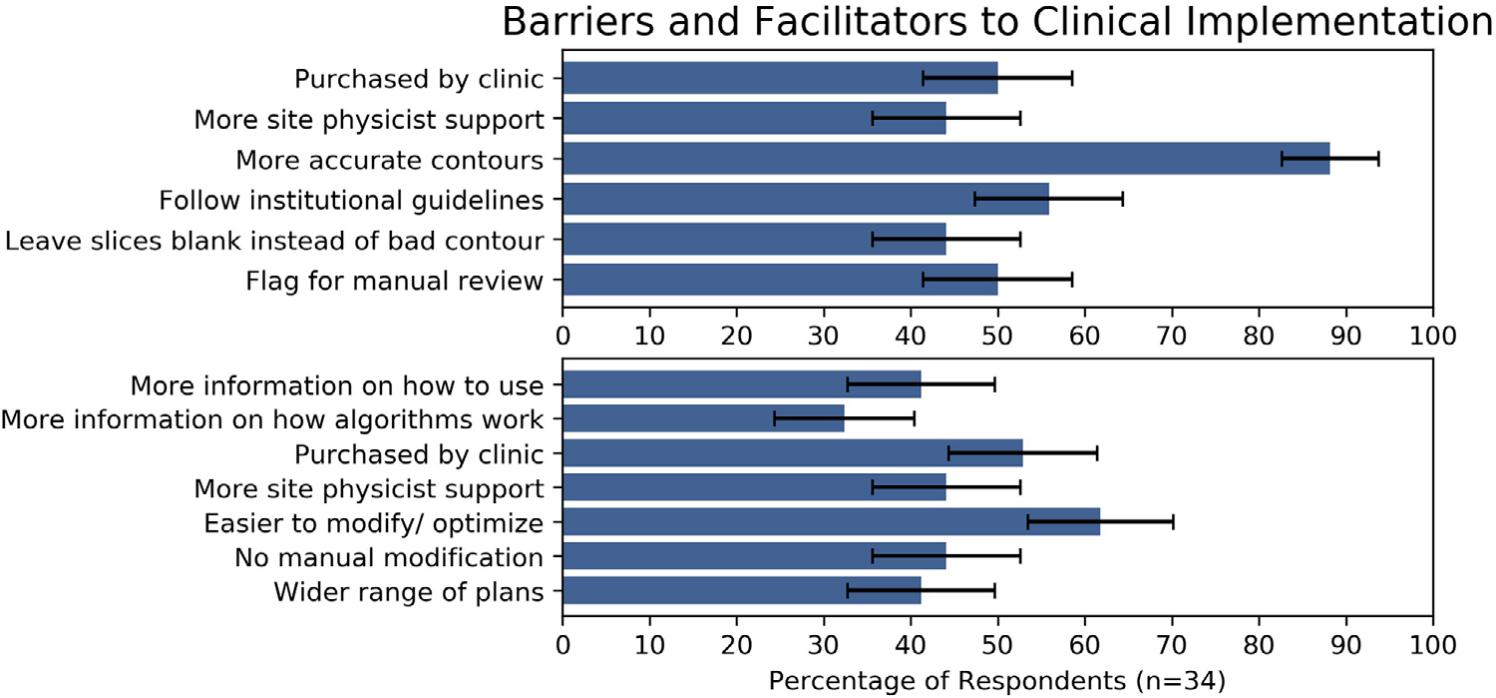
Reported barriers and facilitators to use of auto‐contouring (top) and automated treatment planning (bottom) tools. Error bars represent one standard error

A number of potential barriers and facilitators to use of automation were reported frequently (Figure 2). The most commonly identified barrier to clinical use of AC was contour inaccuracy, with 30 out of 34 survey respondents reporting that increased accuracy would make them more likely to use AC tools. The most commonly identified potential facilitator to use of ATP was if the ATP algorithms would produce plans that were easier to modify in order to get an optimal plan. Twenty‐one out of 34 participants responded this way. However, respondents did see value, or potential value, in both AC and ATP. A significant majority of respondents (23/34) reported liking ATP because it allowed them to work through a higher caseload (Table 3).

**TABLE 3:** Reported reasons for liking/disliking auto‐contouring (AC) and automated treatment planning (ATP)

AC and ATP likes and dislikes (*n* = 34)	Responses (%)
1. Dislike of AC	
Would rather contour from scratch	70.6
Concerned about algorithm making an error	64.7
2. Dislike of ATP	
Do not believe plans are of the same quality	41.2
Takes more time than generating plan from scratch	44.1
Enjoy optimization, do not want to lose that part of job	58.8
3. Like of ATP	
Work through higher patient caseload	67.6
Higher degree of confidence in the plans	29.4

An area of concern for dosimetrists was that the use of automation could increase the likelihood of errors. Among users of deep‐learning‐based and atlas‐based AC, 50% and 52.6%, respectively, were concerned that it could lead to treatment errors. Among users of KBP quality assessment and KBP automated planning, 40% and 38.5%, respectively, were concerned that it would lead to treatment errors. Only 21.4% of users of automated field‐in‐field planning were concerned about errors.

Dosimetrist perceptions of the impact of AC and ATP on job satisfaction and job security appeared to be important. A majority (20/34) reported that they “disliked ATP because they enjoy plan optimization and don't want to give up that part of their job,” and 63.6% agreed or somewhat agreed with the statement that they “value their planning skills highly and would be disappointed to see them devalued.” Likewise, a majority (21/34) agreed or somewhat agreed with the statement “I worry that automated treatment planning will hurt the job market for dosimetrists.” A slight majority (19/34) agreed or somewhat agreed that routinely using ATP could lead to atrophy of planning skills.

No statistically significant correlations were found between AC level of experience (rated on a scale of 1–5) or frequency of use versus level of education, place of employment, and number of machines in the clinic. Similarly, no significant correlation was found between ATP level of experience (rated on the same 1–5 scale) or frequency of use versus these same demographic variables. A statistically significant correlation was found between AC level of experience and view of planning goals at their clinic as standardized (*p* = 0.046), and between ATP level of experience and the same metric of planning goal standardization (*p* = 0.014).

**FIGURE 3:**
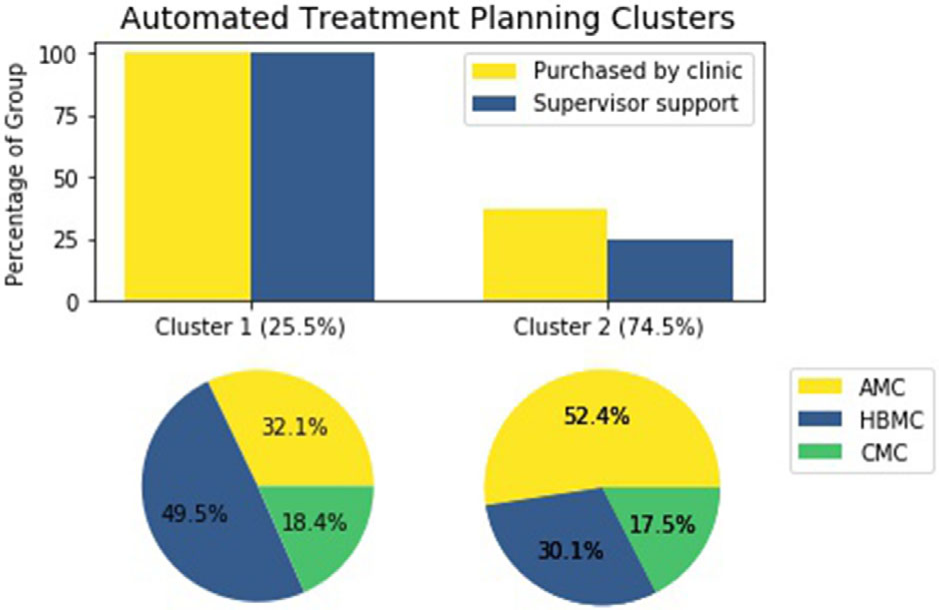
Percentage of dosimetrists reporting certain factors as potential facilitators to use of automated treatment planning by cluster (top); employment breakdown of cluster 1 (bottom left); employment breakdown of cluster 2 (bottom right) Abbreviations: AMC, academic medical center; CMC, community medical center; HBMC, hospital‐based medical center.

Clustering analysis using latent class analysis identified a partition that related to the dosimetrist's clinical environment as a barrier to use for both ATP and AC. A cluster of dosimetrists was identified (comprising 25.5% of respondents overall) that were mainly employed at hospital‐based medical centers (HBMC) or community medical centers (CMC). Hundred percent of participants in this cluster reported being more likely to use ATP if it was both purchased by their clinic and if they received more support from their supervisor or site physicist (Figure 3). This suggests that for dosimetrists employed at non‐academic institutions, lack of access to the technology itself may be an important barrier to use. In the complementary group comprising 74.5% of respondents, the majority reported employment at AMCs. Within this cluster, only 36.8% reported that they would be more likely to use ATP if it was purchased by their clinic, and only 25% reported that they would be more likely to use ATP if they received more support from their site physicist or supervisor. For dosimetrists within this cluster, it appears that access to both ATP tools and support for those tools is a much less significant barrier than for their colleagues at non‐academic institutions.

**FIGURE 4:**
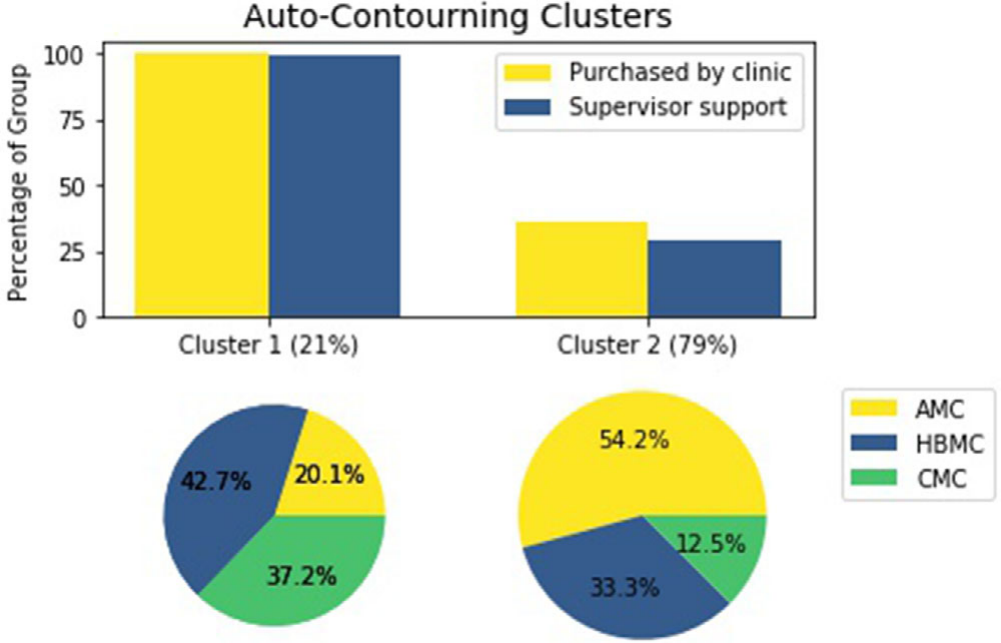
Percentage of dosimetrists reporting certain factors as potential facilitators to use of auto‐contouring by cluster (top); employment breakdown of cluster 1 (bottom left); employment breakdown of cluster 2 (bottom right) Abbreviations: AMC, academic medical center; CMC, community medical center; HBMC, hospital‐based medical center.

An analogous clustering was identified in relation to use of AC with an almost identical group membership. Latent class analysis identified one group (comprising 21% of total respondents) where 20.1% of the cluster reported employment at an AMC, 42.7% reported employment at a HBMC, and 37.2% reported employment at a CMC. For this cluster, 100% of group members reported being more likely to use AC if it was purchased by their clinic while 99.4% reported being more likely to use AC if they received more support from their supervisor or site physicist (Figure 4). In the remaining 79% of respondents, 54.2% reported employment at an AMC, 33.3% at an HBMC, and 12.5% at a CMC. Only 36.7% of this group reported being more likely to use AC if it was purchased by their clinic, and only 29.4% reported being more likely to use AC if they received more support from their supervisor or site physicist. These results again point to lack of access to tools and support for use of these tools as an important barrier for dosimetrists employed in a non‐AMC setting.

## 4 DISCUSSION

This survey has identified three broad barriers to use of automation in treatment planning. The first barrier relates to the limited accuracy and usability, or perception thereof, of the algorithms. A remarkable 30 of 34 respondents thought that AC inaccuracy limited its use. It is noteworthy that a minority of respondents (6/34) reported use of deep‐learning‐based AC, which has been shown in the literature to be significantly more accurate than other methods.^11^, ^38^, ^39^ Therefore, a broader availability of deep‐learning‐based tools could facilitate a broader use of AC in the clinic. A strong majority of respondents thought that it was difficult to modify the output of an automated planning algorithm and this limited the algorithms’ usefulness. This points to human factors engineering^40^ as an important component of treatment planning automation—and of the subsequent clinical implementation—that needs more attention. It should also be noted that the limits of usable accuracy may be lower than what is typically perceived by treatment planners.^41^ Survey results showed that dosimetrists heard about automation most frequently from scientific talks and vendors. Vendors and academic proponents of automated tools may be perceived as biased in their descriptions of algorithm performance. Peer‐to‐peer teaching and continuing medical education focused on automation could address this potential perception gap. Finally, statistically significant correlations were observed between level of experience with ATP and dosimetrists’ perceptions of the degree to which planning goals were standardized at their clinic. This highlights the importance of standardization of clinical goals for the uptake of automation and supports the potential role of automated planning in the context of clinical trials.^42^

The second barrier relates to the perception of the dosimetrist that using automation increases the probability of an error reaching the patient. This directly relates to the well‐documented automation bias.^43^, ^44^ In principle, all the results of treatment planning automation should be reviewed by one or typically more than one human observer. However, little is known about the effectiveness of this review and there is reason to believe it is less than 100% effective.^45^ This points to the need for more research into the effects of automation bias in treatment planning, and if significant, approaches toward minimizing it.

Third, dosimetrists are concerned that treatment planning automation will make their jobs both less satisfying and less secure. A large majority of dosimetrists reported that they enjoyed plan optimization, would not want to lose that part of their job or see it devalued, and expressed explicit job security fears. Contrastingly, in one of the most one‐sided results, 25/34 respondents agreed or somewhat agreed that they would want to use ATP if it worked well. This points to the need for more attention given to developing a picture of what the dosimetrist role looks like as a clinic transitions more fully toward automated technologies.^46^, ^47^ Ultimately, dosimetrists viewed increasing levels of contouring automation as inevitable, with 82.4% agreeing or strongly agreeing that by the end of their career, most or all normal tissue contours will be auto‐generated. A smaller fraction (17/34) believed that by the end of their career, ATP will replace most manual plan optimization.

The results of this survey can be interpreted in light of a technology adoption model such as Venkatesh et al.’s^48^ Unified Theory of Acceptance and Use of Technology (UTAUT). Venkatesh et al. outlined four broad factors that determine how well new technology is taken up within the workplace: performance expectancy, effort expectancy, social influence, and facilitating conditions. The perception of performance of AC tools was negative for most respondents. For ATP, most respondents felt that effort to use was higher than it should be (effort expectancy). Thus, efforts to improve these factors (or the perception of these factors) would be likely to improve adoption of treatment planning automation.

While every effort was made to ensure we obtained a representative sample, we are cognizant of the limitations we faced. Since much of our subject recruitment was conducted via LinkedIn, our sample was weighted to those dosimetrists actively utilizing LinkedIn. We noticed that our sample demographic tended to skew younger than what a truly representative sample would likely show. However, research in this area has shown that statistically correcting for potential demographic biases is not likely to impact the overall conclusions drawn from the data.^49^ Furthermore, the over‐representation of younger dosimetrists in our sample has the potential advantage of offering insight into the factors that will be most relevant to the continuing clinical implementation of automation in radiation oncology, since the majority of the respondents likely expect to continue their employment in this field for decades to come. Finally, there may be a self‐selection process at play because our sample was weighted to responses collected via LinkedIn. These dosimetrists may be more sensitive to or aware of new and emerging technologies in their field and have different perceptions of ATP than their colleagues not using LinkedIn. Every effort was made to emphasize that personal use of ATP was not a prerequisite for our survey, and in our own data we observed responses from dosimetrists ranging from no experience to highly experienced.

In regard to sample size, we acknowledge that our sample size of 34 respondents is not large. We believe some factors mitigate the low absolute number of respondents in our survey. Our survey was detailed, requiring an estimated 15 min to complete, and each response provided a high density of information. Our 34 respondents came from 23 unique institutions (six academic and 17 non‐academic) in California, and survey responses from individual dosimetrists likely represent practice patterns of other dosimetrists at those institutions and correspond to a considerable patient population served. We attempted to reach all medical dosimetrists employed in California by contacting them directly on LinkedIn, and systematically contacting all the chief physicists of non‐AMCs in California who were listed in the AAPM member directory. Of note, no publicly available email directory exists for the American Association of Medical Dosimetrists (AAMD), so we were unable to contact registered dosimetrists in a systematic way via this organization. We were unable to access any internal email address list that might be maintained by the AAMD. Although limited in size, our sample required significant effort to collect and we believe will not be easily surpassed.

Our data may also be limited due to effects from the social desirability bias.^30^ Automation is a new and exciting field, and scores of research has emphasized the benefits of such technological advances.^50^, ^51^ Respondents may have felt pressure to conform to this social bias and offer responses in line with the prevailing opinion of automation as “good.” In our own data, almost all respondents expressed some positive and some negative views of automation. Our survey questions were designed to present both positive and negative views of automation in order to reduce this particular form of bias in the responses.

Response order bias may arise in surveys when respondents process questions in a satisficing instead of optimizing way.^52^ Satisficing respondents are more likely to choose the first reasonable option they are presented with in a list of possible options. However, this effect was not observed in our data. This may be due to our use of “select all that apply” questions to evaluate the underlying attitudes toward automation of our survey sample. The majority of our respondents took the opportunity to select multiple options on these question types, suggesting that they were evaluating each option thoroughly.

Another potential source of bias in our data is due to our use of “agree/disagree” questions to measure respondents’ views of and attitudes toward automation. These questions can pose challenges through a tendency for respondents to initially agree with the assertion being made in the statement and spend more time looking for reasons to agree with the statement than looking for reasons to disagree.^30^ In order to minimize this bias, we balanced level of agreement statements with positive and negative views of automation. We observed approximately equal numbers of agreement and disagreement, indicating a low degree of agreement bias in our results.

## 5 CONCLUSION

To our knowledge this is the first systematic investigation into the views of automation by medical dosimetrists, who perform the majority of treatment planning at many if not most radiotherapy facilities. We have explicitly identified potential barriers and facilitators to use of automated technologies in the radiation therapy treatment planning workflow. This investigation highlights several concrete approaches that could potentially increase the translation of treatment planning automation into the clinic, as well as areas of needed research.

## AUTHOR CONTRIBUTION

Rachel Petragallo designed the survey and collected survey results, performed the statistical analysis, and drafted the manuscript. Naomi Bardach and Ezequiel Ramirez participated in survey design and editing of the manuscript. James M. Lamb conceptualized and provided overall direction to the study and participated in survey design, data collection and editing of the manuscript.

## CONFLICT OF INTEREST

The authors have no conflict of interest to report.

## Supporting information

SUPPORTING INFORMATION

## Data Availability

Research data will be shared to the extent allowed under Institutional Review Board approval upon request to the corresponding author.
