# Supplementary material for: Barriers and facilitators to clinical implementation of radiotherapy treatment planning automation: A survey study of medical dosimetrists
Source: J Appl Clin Med Phys. 2022 Mar 3;23(5):e13568. doi: 10.1002/acm2.13568 (PMC9121037; doi:10.1002/acm2.13568)
Supplement: Supplementary file 1 — SUPPORTING INFORMATION [file ACM2-23-e13568-s001.docx]

**Prior Use:**

1. I have used the following types of automation tools for radiation oncology (select all that apply). **Please select all options that you have used at any point in your career**, even if you do not currently use that tool or if you have used it infrequently.

- Deep learning-based auto-contouring (for example, Mirada DLCExpert, MIM ContourProtege-AI)
- Atlas-based and/or model-based auto-contouring algorithms (for example, MIM Atlas Segment, Varian Velocity, RayStation MABS/MBS, Pinnacle SPICE, Elekta ABAS)
- Knowledge-based plan quality assessment (for example, Sun Nuclear PlanIQ)
- Automated planning using knowledge-based planning (KBP) algorithms (for example, Varian RapidPlan)
- Automated field-in-field planning (for example, Radformation EZFluence)
- Automated planning using rule-based or template-based algorithms (for example, Phillips Pinnacle Auto-Planning, Raysearch Raystation Auto-Planning)
- Automated planning using other algorithm not listed above
- Automated planning, specific algorithm unknown

1b. If you selected “other algorithm”, please specify the algorithm used in the field below:

**Auto-Contouring:**

2. Please rate your level of experience with auto-contouring from 1 (not familiar at all) to 5 (extremely familiar)

- 1
- 2
- 3
- 4
- 5

3. I have used deep learning-based auto-contouring (e.g. Mirada DLCExpert, MIM ContourProtege-AI) for the following body sites (select all that apply):

- Head and neck
- Thorax
- Breast
- Pelvis (prostate, bladder, rectal, etc.)
- Extremities
- Intra-cranial
- None of the above

4. I have used atlas-based and/or model-based auto-contouring (e.g. MIM Atlas Segment, Varian Velocity, RayStation MABS/MBS, Pinnacle SPICE, Elekta ABAS) for the following body sites (select all that apply):

- Head and neck
- Thorax
- Breast
- Pelvis (prostate, bladder, rectal, etc.)
- Extremities
- Intra-cranial
- None of the above

5. How often do you use auto-contouring?

- Daily
- Weekly
- Once or twice a month
- Less than once a month
- Never

6. Please rank the following options to indicate where you have heard about auto-contouring. Enter 1 for where you’ve heard about it the most and 4 for where you’ve heard about it the least.

- Scientific talks at professional meetings
- Vendor booths at professional meetings
- Peers at other clinics
- Colleagues at my own workplace

7. I would be more likely to use auto-contouring algorithms if (select all that apply):

- My clinic purchased an auto-contouring product
- My site physicist and/or supervisor provided more support of auto-contouring
- Auto-contouring algorithms were more accurate.
- Auto-contouring algorithms could produce contours that followed my institution’s contouring guidelines.
- Auto-contouring algorithms would leave image slices blank rather than producing a contour in those slices that then needed to be heavily edited
- Auto-contouring algorithms could tell me specific parts of the contour that needed my attention
- Other

7b. Please specify other things that would make you more likely to use auto-contouring algorithms in the field below:

8. I dislike auto-contouring because (select all that apply):

- I would rather start contouring from scratch than have to modify auto-contours, even if the time taken is about the same
- I am concerned that the algorithm will make a contouring error that I won’t catch
- Other

8b. Please specify other reasons you dislike auto-contouring in the field below:

9. Please rate your level of agreement with the following statements based on your personal experience using **deep learning-based auto-contouring** (e.g. Mirada DLCExpert, MIM ContourProtege-AI). If you feel that the statement does not apply to your personal experience, select “not relevant to me.”

Options: disagree, somewhat disagree, neither agree nor disagree, somewhat agree, agree, not relevant to me

- Modifying auto-contours takes longer than creating the contours from scratch
- The contours produced by the deep learning-based algorithm are often so incorrect that auto-contouring adds nothing of value
- Auto-contouring saves enough time for me to think that it’s worth using
- I am concerned that use of auto-contouring could lead to treatment errors; for example if the algorithm contours an organ incorrectly and leads to an overdose of that organ
- I believe that deep learning-based auto-contouring is ready for routine clinical use
- When I use deep learning-based auto-contouring, I spend a lot of time checking the contours produced by the algorithm

10. Please rate your level of agreement with the following statements based on your personal experience using **atlas-based and/or model-based auto-contouring** (e.g. MIM Atlas Segment, Varian Velocity, RayStation MABS/MBS, Pinnacle SPICE, Elekta ABAS). If you feel that the statement does not apply to your personal experience, select “not relevant to me.”

Options: disagree, somewhat disagree, neither agree nor disagree, somewhat agree, agree, not relevant to me

- Modifying auto-contours takes longer than creating the contours from scratch
- The contours produced by the atlas-based/ model-based algorithm(s) are often so incorrect that auto-contouring adds nothing of value
- Auto-contouring saves enough time for me to think that it’s worth using
- I am concerned that use of auto-contouring could lead to treatment errors; for example if the algorithm contours an organ incorrectly and leads to an overdose of that organ
- I believe that atlas-based/ model-based auto-contouring is ready for routine clinical use
- When I use atlas-based/ model-based auto-contouring, I spend a lot of time checking the contours produced by the algorithm

**Automated Treatment Planning:**

11. Please rate your level of experience with automated treatment planning **in general** from 1 (not familiar at all) to 5 (extremely familiar)

- 1
- 2
- 3
- 4
- 5

12. I have used knowledge-based plan quality assessment (e.g. Sun Nuclear PlanIQ) for the following body sites (select all that apply):

- Head and neck
- Thorax
- Breast
- Pelvis (prostate, bladder, rectal, etc.)
- Extremities
- Intra-cranial
- None of the above

13. I have used automated planning using KBP algorithms (e.g. Varian RapidPlan) for the following body sites (select all that apply):

- Head and neck
- Thorax
- Breast
- Pelvis (prostate, bladder, rectal, etc.)
- Extremities
- Intra-cranial
- None of the above

14. I have used automated field-in-field planning (e.g. Radformation EZFluence) for the following body sites (select all that apply):

- Head and neck
- Thorax
- Breast
- Pelvis (prostate, bladder, rectal, etc.)
- Extremities
- Intra-cranial
- None of the above

15. I have used automated planning using rule-based or template-based algorithms (e.g. Phillips Pinnacle Auto-Planning, Raysearch Raystation Auto-Planning) for the following body sites (select all that apply):

- Head and neck
- Thorax
- Breast
- Pelvis (prostate, bladder, rectal, etc.)
- Extremities
- Intra-cranial
- None of the above

16. I have used automated treatment planning for the following body sites (select all that apply):

- Head and neck
- Thorax
- Breast
- Pelvis (prostate, bladder, rectal, etc.)
- Extremities
- Intra-cranial
- None of the above

17. How often do you use automated treatment planning?

- Daily
- Weekly
- Once or twice a month
- Less than once a month
- Never

18. I would be more likely to use automated treatment planning if (select all that apply):

- I was provided with more information about how to use the tools
- I was provided with more information about how the algorithms work behind the scenes
- My clinic purchased an automated treatment planning product
- My site physicist and/or supervisor provided more support and/or more training on automated treatment planning
- The automated treatment planning algorithm produced a plan that was easier to modify or “tweak” to get the optimal plan
- The automated treatment planning algorithm produced better plans that didn’t need any modifications by the dosimetrist
- Automated treatment planning was available for a wider range of types of treatment plans
- Other

18b. Please specify other things that would make you more likely to use automated treatment planning in the field below:

19. I like automated treatment planning because (select all that apply):

- It allows me to work through a higher patient caseload
- I have a higher degree of confidence in the quality of the plans that I am submitting to the prescribing physician
- Other

19b. Please specify other reasons that you like automated treatment planning in the field below:

20. I dislike automated treatment planning because (select all that apply):

- I do not believe that the plans are of the same quality as those generated by experienced dosimetrists
- Modifying automated plans takes more time than generating a comparable quality plan from scratch
- I enjoy plan optimization and I don’t want to give up that part of my job
- Other

20b. Please specify other reasons that you dislike automated treatment planning in the field below:

21. Please rate your level of agreement with the following statements based on your personal experience using **knowledge-based plan quality assessment** (e.g. Sun Nuclear PlanIQ). If you feel that the statement does not apply to your personal experience, select “not relevant to me.”

Options: disagree, somewhat disagree, neither agree nor disagree, somewhat agree, agree, not relevant to me

- Knowledge-based plan quality assessment leads to higher quality treatment plans
- Knowledge-based plan quality assessment saves time by helping me know when my plan is good enough so that I can stop optimizing
- I am concerned that use of knowledge-based plan quality assessment could lead to treatment errors; for example if the algorithm makes an error and I don’t catch it.
- Knowledge-based plan quality assessment decreases the amount of time I spend on any single patient case.
- I believe that knowledge-based plan quality assessment is ready for routine clinical use.

22. Please rate your level of agreement with the following statements based on your personal experience using **KBP automated treatment planning** (e.g. RapidPlan). If you feel that the statement does not apply to your personal experience, select “not relevant to me.”

Options: disagree, somewhat disagree, neither agree nor disagree, somewhat agree, agree, not relevant to me

- KBP automated planning leads to higher quality treatment plans
- KBP automated planning saves time by helping me know when my plan is good enough so that I can stop optimizing
- I am concerned that use of KBP automated planning could lead to treatment errors; for example if the algorithm makes an error and I don’t catch it.
- KBP automated planning decreases the amount of time I spend on any single patient case.
- I believe that KBP automated planning is ready for routine clinical use.

23. Please rate your level of agreement with the following statements based on your personal experience using **automated field-in-field planning** (e.g. EZFluence). If you feel that the statement does not apply to your personal experience, select “not relevant to me.”

Options: disagree, somewhat disagree, neither agree nor disagree, somewhat agree, agree, not relevant to me

- Automated field-in-field planning leads to higher quality treatment plans
- I am concerned that use of automated field-in-field planning could lead to treatment errors; for example if the algorithm makes an error and I don’t catch it.
- Automated field-in-field planning decreases the amount of time I spend on any single patient case.
- I believe that automated field-in-field planning is ready for routine clinical use.

24. Please rate your level of agreement with the following statements based on your personal experience using **automated treatment planning using rule-based or template-based algorithms** (e.g. Pinnacle Auto-Planning, RayStation Auto-Planning). If you feel that the statement does not apply to your personal experience, select “not relevant to me.”

Options: disagree, somewhat disagree, neither agree nor disagree, somewhat agree, agree, not relevant to me

- The automated treatment planning tool I have used leads to higher quality treatment plans
- I am concerned that use of automated treatment planning could lead to treatment errors; for example if the algorithm makes an error and I don’t catch it.
- Automated treatment planning decreases the amount of time I spend on any single patient case.
- I believe that the automated treatment planning tool I have used is ready for routine clinical use.

25. Please rate your level of agreement with the following statements based on your personal experience using automated treatment planning. If you feel that the statement does not apply to your personal experience, select “not relevant to me.”

Options: disagree, somewhat disagree, neither agree nor disagree, somewhat agree, agree, not relevant to me

- The automated treatment planning tool I have used leads to higher quality treatment plans
- I am concerned that use of automated treatment planning could lead to treatment errors; for example if the algorithm makes an error and I don’t catch it.
- Automated treatment planning decreases the amount of time I spend on any single patient case.
- I believe that the automated treatment planning tool I have used is ready for routine clinical use.

**General level of Agreement:**

26. Please rate your level of agreement with the following statements:

Options: disagree, somewhat disagree, neither agree nor disagree, somewhat agree, agree, not relevant to me.

- I believe auto-contouring will continue to get better and by the end of my career, most or all normal tissue contours will be automatically generated.
- I worry that automated treatment planning will hurt the job market for dosimetrists.
- If automation reduces the time to make a plan, I will just get more plans and be even busier.
- I worked hard to gain my treatment planning skills and I value them highly. To see them devalued would be a disappointment.
- I am concerned that routinely using automated treatment planning will cause me to get out of practice on planning difficult patient cases.
- I believe automated treatment planning algorithms will continue to get better and by the end of my career will replace most manual treatment plan optimization.
- Planning goals at my clinic aren’t very standardized.
- I would want to use automated treatment planning tools if I knew they worked well.

**Demographics:**

27. What is your age?

- 20-29
- 30-39
- 40-49
- 50-59
- 60+
- Prefer not to answer

28. How many years have you been employed as a medical dosimetrist?

- Less than 5
- 5-9
- 10-19
- 20+

29. Are you certified by the Medical Dosimetrist Certification Board?

- Yes
- No

30. What is your gender?

- Male
- Female
- Other/ non-binary
- Prefer not to answer

31. What is your highest level of education?

- Associate’s degree
- Bachelor’s degree
- Master’s degree
- Doctorate
- Prefer not to answer

32. Which of the following best describes your current clinical environment?

- Non hospital-based community practice
- Hospital-based non-academic medical center
- Academic medical center

33. How many radiotherapy treatment machines are in use at your clinic (including any satellite clinics)?

- 1
- 2-4
- 5-8
- 9+
